# Influence of Allee effect on the spatiotemporal behavior of a diffusive predator–prey model with Crowley–Martin type response function

**DOI:** 10.1038/s41598-023-28419-0

**Published:** 2023-03-22

**Authors:** Lakshmi Narayan Guin, Pallav Jyoti Pal, Jawaher Alzahrani, Nijamuddin Ali, Krishnendu Sarkar, Salih Djilali, Anwar Zeb, Ilyas Khan, Sayed M Eldin

**Affiliations:** 1 Department of Mathematics, Visva-Bharati, Santiniketan, West Bengal 731235 India; 2 Department of Mathematics, Krishna Chandra College, Hetampur, Birbhum, West Bengal 731124 India; 3 Department of Mathematics, College of Education, Majmmah University, Al-Majmaah, 11952 Saudi Arabia; 4 Department of Mathematics, Vivekananda Mahavidyalaya, Purba Bardhaman, West Bengal 713103 India; 5 Laboratoire d’Analyse Non Linéaire et Mathématiques Appliquées, University of Tlemcen, Tlemcen, Algeria; 6 Department of Mathematics, Universite Hassiba Benbouali de Chlef, 02000 Chlef, Algeria; 7 Department of Mathematics, COMSATS University Islamabad, Abbottabad, Khyber Pakhtunkhwa 22060 Pakistan; 8 Department of Mathematics, College of Science Al-Zulfi, Majmaah University, Al-Majmaah, 11952 Saudi Arabia; 9 Center of Research, Faculty of Engineering, Future University in Egypt, New Cairo, 11835 Egypt

**Keywords:** Applied mathematics, Chemical engineering

## Abstract

The present paper is dealt with a predator–prey model in which the growth of the prey population is influenced by the Allee effect while the predator species are contended with the prey population following the Crowley–Martin type response function. The proposed model is comprehensively analyzed in terms of stability and manifestation of bifurcation of the system. The system unveils the bi-stability together with the existence of a separatrix. In view of the eminence of spatial ecology, the dynamical complexity emanating from the induction of the Allee effect in prey species of a Crowley–Martin reaction–diffusion predator–prey model is also investigated profoundly. The results of numerical simulations reveal that the present system dynamics is motivated by both the Allee effect and diffusion-controlled pattern formation growth to hot spots, stripe-hot spot mixtures, stripes, labyrinthine, stripe-cold spot mixtures, and cold spots replication. The theoretical consequences of the spatiotemporal model under study are validated through numerical simulations.

## Introduction

During the last couple of decades, the Allee effect, together with its immediate consequences on ecology and conservation of biomass has been drawing attention to the researchers^1,2^. The Allee effect usually refers to a process that alleviates the growth rate for small population density^2^. For example, the Allee effect in prey population is designated by the fact that the prey species possess a probability of localized extinction, causing the specialist predator to suffer from looking for resources thereby. This may result in saturation in consumption that goes against the assumption in the realm of ecological system^3–6^. The concept of the Allee effect was widely accepted and gained momentum in major recognition towards the golden period of the late eighties of the 20th century^7–11^. It has been reported that the induction of the Allee effect in the predator–prey interacting system leads to various dynamical complexities^12–16^ where the researchers highlighted mainly on the conditions for extinction and survival of the population. Diverse factors, including mating limitation, cooperative defense, combined feeding, and environmental conditioning, give rise to the growth of prey species influenced by the Allee effect. Of all these factors, the mating constraint^17^ seems to be the most pervasive one as it becomes difficult to locate the mating individuals at low density.

In the event of one, multi Allee effects exerting impact on a single population concomitantly, the situation may be expressed as single, double, or multiple Allee effects, respectively. It can be either multiplicative or additive. The most known increasing function that describes this effect is

1 $$\begin{aligned} \frac{dx}{dt}=rx\left( 1-\frac{x}{K}\right) (x-l), \end{aligned}$$

where *x* is the density of the population sample, *K* is the carrying capacity, *r* is the instinct birth rate. The term $(x-l)$ is considered as a modification of the classical logistic model:

2 $$\begin{aligned} \frac{dx}{dt}=rx\left( 1-\frac{x}{K}\right) , \end{aligned}$$

It is not difficult to show that for $x<l$ the population density will extinct, while if $x>l$ the population grows to *K*. Allee effect expressed by Eq. (1) is strong or weak according to $l>0$ (*l* is the threshold population level) or $-K<l\le 0$ respectively. For more examples of modeling Allee effect we cite the research^12,16,18^.

In the present perspective, the functional response corresponds to the predator’s per capita feeding rate on prey. Plenty of functional responses are available in the ecological literature, and all of them have contributed much to enriching the behaviour of the predator–prey system depending on the objectives of the problems. In ecology, the functional response can be affected by prey habitat, prey escaping ability, and predator hunting ability^19^. Two types of functional responses are often used-one is prey-dependent and other is predator-dependent. The former is a function of prey species only while the later is a function of the two populations. Lotka–Volterra type, Holling type II, III, IV, and Ivlev type are prey-dependent functional responses of which Holling II interaction functional is the most common functional response in nature^20–22^. On the other hand, ratio-dependent function (singular at origin), Hassell–Varley, Crowley–Martin, and Beddington–DeAngelis are the predator-dependent functional responses^15,22–25^. Considering the complexity of interactions among species, it is more realistic that the interaction functional depends not prey population only but also on the predator species^26^. Experimental studies on Dragonfly, carried out by Crowley and Martin^24,26^ revealed a new functional response that is defined by

3 $$\begin{aligned} f(X,Y)=\frac{MX}{1+AX+BY+ABXY}, \end{aligned}$$

where the parameters *M*, *A* and *B* are positive depicting the impacts of capture rate, handling time and the size of obstructions among predators, individually, on the feeding rate. In case $A = 0$, $B = 0$, at that point Crowley–Martin interaction functional becomes Holling I interaction functional; on the off chance that $A>0$, $B =0$, at that point Crowley–Martin interaction functional reduces to Holling II interaction functional; when $A = 0$, $B > 0$, it reduces to Harrison interaction functional^27^. Since the functional response under consideration is quite general, encompassing several cases mentioned above, it is essential to study ecological interactions with the Crowley–Martin interaction functional^28–30^. Crowley–Martin interaction functional is utilized for information sets that show an asymptotic nourishing rate influenced by predator density^26^. Particularly, in a recent article^31^, authors mentioned that the predator-dependent, asymptotic interaction functional models (that is, Hassell–Varley sort II and Crowley–Martin) performed best among a set of 23 competing direct, asymptotic and sigmoid models. Although, as it were, some models with the Allee effect and predator-dependent reaction work have been explored to date^15,16,32,33^.

The present investigation is attached to the Allee effect, which is currently an exciting and challenging research topic in population dynamics. Consequently, many eminent researchers investigated the Allee effect scenario theoretically and experimentally. In mathematical ecology, one may understand the Allee effect scenario as a decline in individual fitness at low species concentration or population size, resulting in critical population thresholds below which species crash to extinction^7^??. The influence of Allee effect plays an important role in ecological studies (namely, conservation; the management of endangered species; biological invasions; pest control, etc.) since the Allee effect can greatly increase the likehood of local and global extinction. Individuals in a population be unsuccessful to locate an appropriate mate during their reproductive period at low density, thus resulting in reduced reproductive outputs, and then a mate-finding Allee effect may arise. Examples include the Glanville fritillary butterfly, sheep ticks, and whales^10^. Modern literature survey related to the appearance or application areas of the Allee effect in ecological modelling is provided below:

1. Allee effect initiates with reproductive mechanisms, as well as fertilization efficiency in broadcast spawners. For instance, eggs of aquatic animals (oysters, fishes etc.) that lay many small eggs;
2. Limitation of spermatozoon, reproductive facilitation by conspecific and female selection;
3. Pollen limitation and mate searching;
4. Survival mechanisms: environmental conditioning and mainly predation such as flocking, colonialist, and group vigilance;
5. Allee effect is significant for social and cooperative species, where group size plays a vital role in reproduction and survival.

In 2016, Rocha et al. explained the unstable scenario (via bifurcation and extinction cases) of interacting species with a weak Allee effect, where the growth follows Richards’ growth law?. The parametric conditions for stability and the region of the positive solution have been offered for fishery and genetic models with the Allee effect??. In the proposed research, our prime objective is to suggest the consequence of the Allee effect in the reaction–diffusion equation and confirm how it affects interacting species in ecology. We investigate the impact of the multiplicative Allee effect in presence of self-diffusion for a small or sparse population.

In 1952, Turing^34^ presented a dynamical behavior, which has been broadly utilized to create a clear view of how Turing designs are molded, and it is well-known as Turing bifurcation or Turing instability. Recently, reaction–diffusion frameworks have expanded concentration from Mathematical ecologists to look for experiences into the intriguing spatial patterns that take place in living life forms and in environmental frameworks. The conduct of spatiotemporal patterns and the affect of spatial diffusion on the species is an important topic and the subject of discussion in mathematical ecology and biology, science, and intuitive biochemical^35–40^. The spatial factor of ecological communications has been documented as an indispensable factor in determining how populations are formed and addressing the challenging function of space, both theoretical and experimental^37^. Empirical proof recommends that the spatial scale and structure of the environment can impact populace intelligent^41^. For more methods of natural modelling we cite the researches^42–46^.

The present research is structured as follows. The next section is put to study the formulation of the Allee model undertaken along with the stated assumptions. “Mathematical analysis” contains the mathematical analysis of the investigated model, where the existence of the equilibrium states is proved and their stability for the non-diffusive system. For the diffusive system, we demonstrate the influence of spatial diffusion on the stability of the equilibria. Numerical simulation has finally been carried out in “Numerical simulations”. The investigation concludes by discussing the results obtained in “Discussion and conclusion”* finally.

## Mathematical model

The general predator–prey model formulated by differential equations can be structured as

4a $$\begin{aligned}{} & {} \frac{dX}{dT}=Xg(X, K)-Yf(X, Y), \end{aligned}$$

4b $$\begin{aligned}{} & {} \frac{dY}{dT}=EYf(X, Y)-DY, \ \end{aligned}$$

with *X*(*T*) and *Y*(*T*) are respectively the sizes of prey population and predator population; *f*(*X*, *Y*) is the interaction functional; *E* is the resources utilization rate ($0<E<1$); *D* is the predators death rate. *g*(*X*, *K*) is growth function of *X*. Considering $g(X, K)=RX\left( 1-\frac{X}{K}\right) (X-l)$ as Eq. (1) and functional response of predators *f*(*X*, *Y*) as Beddington–DeAngelis functional response given by $\frac{MX}{1+AX+BY}$, a predator–prey system can be prepared as

5a $$\begin{aligned}{} & {} \frac{dX}{dT}=RX\left( 1-\frac{X}{K}\right) (X-L)-\frac{MXY}{1+AX+BY}, \end{aligned}$$

5b $$\begin{aligned}{} & {} \frac{dY}{dT}=\frac{EMXY}{1+AX+BY}-DY, \nonumber \\{} & {} X(0) \ge 0,\quad Y(0) \ge 0. \end{aligned}$$

Although, in comparison with the Beddington–DeAngelis functional response, Crowley–Martin has an additional product term in its denominator, namely *ABXY* that models common impedances among predators. The Beddington–DeAngelis interaction functional presumes that dealing with and looking are commonly elite, that’s predators dealing with prey will not meddle with those looking for prey. On the other hand, Crowley–Martin utilitarian reaction permits for impedances among predators indeed in case they are taking care of prey^24^. Hence the environmental demonstration with Crowley–Martin sort useful reaction advances to the Beddington–DeAngelis demonstrate.

In the present investigation, we investigate a predator–prey model where the prey growth rate is subject to an Allee effect next to a Crowley–Martin type interaction functional which is given by (3). The species interactions are described by the following system of ODEs

6a $$\begin{aligned}{} & {} \frac{dX}{dT}=RX\left( 1-\frac{X}{K}\right) (X-L)-\frac{MXY}{1+AX+BY+ABXY}, \end{aligned}$$

6b $$\begin{aligned}{} & {} \frac{dY}{dT}=\frac{EMXY}{1+AX+BY+ABXY}-DY, \nonumber \\{} & {} \quad X(0) \ge 0,\quad Y(0) \ge 0. \end{aligned}$$

Basically, we explore a deterministic continuous two-dimensional interacting species model under the presumptions that species size changes by time only. The patterns of diffusive Holling–Tanner predator–prey model has confirmed quite motivating and obtained increasing interest by both mathematicians and ecologists in many research papers. By considering the importance of spatial ecology, we introduce diffusion effect in the system (6). Hence, the system (6) becomes a diffusive predator–prey model equations:

7a $$\begin{aligned} \frac{\partial X}{\partial T}= & {} RX\left( 1-\frac{X}{K}\right) (X-L)-\frac{MXY}{1+AX+BY+ABXY} + D_{1}\nabla _{1}^{2}X, \end{aligned}$$

7b $$\begin{aligned} \frac{\partial Y}{\partial T}= & {} \frac{EMXY}{1+AX+BY+ABXY}-DY + D_{2}\nabla _{1}^{2}Y, \end{aligned}$$

7c $$\begin{aligned} X(0, \xi _{1}, \eta _{1})\ge & {} 0, \; \; Y(0, \xi _{1}, \eta _{1}) \ge 0,\end{aligned}$$

7d $$\begin{aligned} \frac{\partial X}{\partial \varvec{n}}= & {} \frac{\partial Y}{\partial \varvec{n}}=0,\;\;(\xi _{1}, \eta _{1}) \in \partial \Omega , \end{aligned}$$

where $X(0, \xi _{1}, \eta _{1})$ and $Y(0, \xi _{1}, \eta _{1})$ stands for the prey and predator population densities at any time *T* and at the spatial location $(\xi _{1}, \eta _{1})$ in 2*D* spatial domain; $\nabla _{1}^{2} \equiv \frac{\partial ^{2}}{\partial \xi _{1}^{2}}+\frac{\partial ^{2}}{\partial \eta _{1}^{2}}$ is the Laplacian operator in 2*D* spatial domain; $\varvec{n}$ is the outward unit normal vector of the smooth boundary $\partial \Omega$ of the reaction–diffusion domain $\Omega$, remained parameters have the same biological relevance as in (6). Throughout the investigation, one we can consider the homogeneousness of the environment.

The resulting framework may be a two-component coupled nonlinear partial differential equations (PDEs). For the proposed reaction–diffusion model $D_{1}$ and $D_{2}$ are the positive self-diffusion rates of the prey and the predator densities, respectively. Moreover, self-diffusion is measured like a spatial transmission approach, which goes from higher concentration to lower one. The last line in (7) is the homogeneous Neumann boundary condition which demonstrates that the framework is self-contained with zero population flux over the bounders.

The salient motivation of the current investigation is to make the proposed model more realistic subject to the appropriate ecological influences. Throughout the research manuscript, we try to address the following questions in a usual way: What happens if one takes into account the combined effect of the multiplicative Allee effect and self-diffusion? What does the impact of the multiplicative Allee effect and diffusion-induced spatial pattern of the system? These assist in motivating the concerned researcher to pretense the following imperative and motivating questions:

1. Can the multiplicative Allee factor destabilize any coexistence equilibrium point of the model system through Hopf bifurcation in the corresponding temporal environment in the presence of Crowley–Martin functional response?
2. Does the multiplicative Allee factor still play a key role in the mechanism for destabilizing the spatiotemporal predator–prey system in the presence of a positive diffusion coefficient? Has any special impact for the diffusion-based restriction $d_{2}>d_{1}$?
3. Can the multiplicative Allee factor control the spatially inhomogeneous distribution of species employing diffusion-driven instability? If yes, how to prepare for spatial pattern transition to explain populations’ spatially inhomogeneous distribution? What about other system parameters of the proposed model system?

However, so far our knowledge goes, no work has been carried out in this direction by considering the combined effect of the multiplicative Allee factor and self-diffusion in an interacting species system in a spatiotemporal environment. With this particular incentive, the current investigation considers the Crowley–Martin predator–prey interaction with the self-diffusion structure. To address the questions **(Q1)–(Q3)** properly, we have focused our attention on the collective impact among multiplicative Allee factor and diffusion-induced spatiotemporal pattern in the proposed model system.

## Mathematical analysis

Here, we are interested in analyzing mathematically the system (6) and (7). At first, for the purpose of reducing the number of parameters we consider the change of variable $x=\frac{X}{K}, y=\frac{Y}{KE}, t=KRT$ and model (6) becomes

8a $$\begin{aligned}{} & {} \frac{dx}{dt}=x\left( 1-x\right) (x-l)-\frac{\beta xy}{1+ax+by+abxy}, \end{aligned}$$

8b $$\begin{aligned}{} & {} \frac{dy}{dt}=\frac{\beta xy}{1+ax+by+abxy}-dy, \end{aligned}$$

8c $$\begin{aligned}{} & {} x(0) \ge 0,\quad y(0) \ge 0, \end{aligned}$$

with $l=\frac{L}{K}$, $\beta = \frac{ME}{R}$, $a=AK$, $b=BKE$, $d=\frac{D}{KR}$. Similarly, for the diffusive system we introduce the change of variables: $x=\frac{X}{K}, y=\frac{Y}{KE}, t=KRT, \xi =\frac{\xi _{1}}{L_{1}}, \eta =\frac{\eta _{1}}{L_{1}}$, $L_{1}$ is the characteristic length of the 2*D* spatial domain $\Omega$; and the model (7) becomes

9a $$\begin{aligned} \frac{\partial x}{\partial t}= & {} x\left( 1-x\right) (x-l)-\frac{\beta xy}{1+ax+by+abxy} + d_{1}\nabla ^{2}x, \end{aligned}$$

9b $$\begin{aligned} \frac{\partial y}{\partial t}= & {} \frac{\beta xy}{1+ax+by+abxy}-dy + d_{2}\nabla ^{2}y, \end{aligned}$$

9c $$\begin{aligned} x(0, \xi , \eta )\ge & {} 0, \; \; y(0, \xi , \eta ) \ge 0, \end{aligned}$$

where $(\xi , \eta ) \in \Omega$; $\Omega \subseteq {\textbf {R}}^{2}$ is the 2*D* bounded connected square domain in ${\textbf {R}}$ with smooth boundary $\partial \Omega$; $l=\frac{L}{K}$, $\beta = \frac{ME}{R}$, $a=AK$, $b=BKE$, $d=\frac{D}{KR}$, $d_{1}=\frac{D_{1}}{RKL_{1}^{2}}$, $d_{2}=\frac{D_{2}}{RKL_{1}^{2}}$ and $\nabla ^{2} \equiv \frac{\partial ^{2}}{\partial \xi ^{2}}+\frac{\partial ^{2}}{\partial \eta ^{2}}$ in 2*D* spatial domain $\Omega$. Note that, since (9) does not take into consideration the conceivable presence of alternative nourishment sources, predators cannot survive without prey, and prey extinction leads to the extinction of both species. In addition, the homogeneous Neumann boundary condition becomes:

$$\begin{aligned} \frac{\partial u}{\partial \varvec{n}}=\frac{\partial v}{\partial \varvec{n}}=0,\;\;(\xi , \eta ) \in \partial \Omega , \end{aligned}$$

### Homogeneous states

The equilibrium states for (9) are the intersection points points the two curves:

10a $$\begin{aligned}{} & {} x\left( 1-x\right) (x-l)=\frac{\beta xy}{1+ax+by+abxy}, \end{aligned}$$

10b $$\begin{aligned}{} & {} \frac{\beta xy}{1+ax+by+abxy}=dy, \ \end{aligned}$$

**Figure 1:**
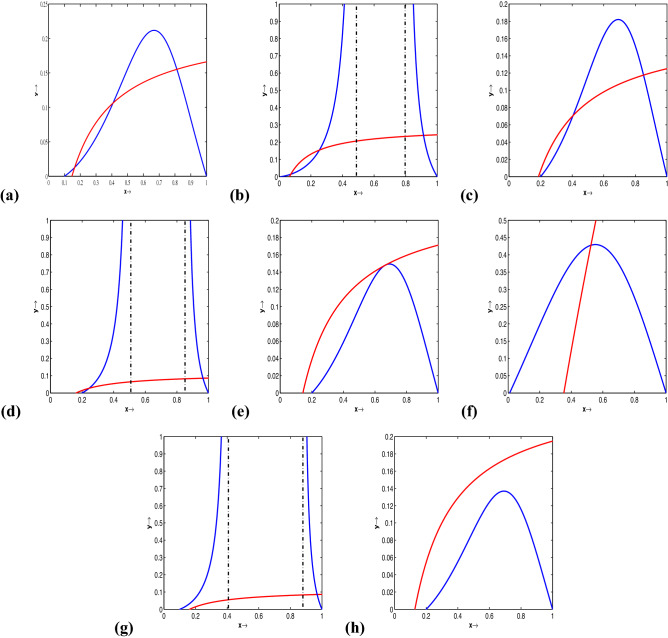
(Colour online) Strong Allee effect: prey-nullclines are identified by solid blue curves and, accordingly, predator-nullclines are recognized by solid red curves. (**a**) For $\beta =10$,the two nullclines cross thrice, then there exist three positive intersections. (**b**) For $l= 0.01, \beta =7.8, a = 10.5, b = 5.2, d = 0.3$, in this case we have three interior equilibrium points. (**c**) Two equilibrium points exists for $l= 0.2; \beta =9; a = 7.5; b = 4.1; d = 0.7$. (**d**) For $l= 0.2, \beta =5, a = 10.5, b = 5.2, d = 0.3$, in this case we obtain the existence of two equilibrium points. (**e**) one equilibrium point exists for $l= 0.2; \beta =10.1267155062337; a = 7.5; b = 4.1; d = 0.7$, (**f**) one equilibrium point for $l= 0.01, \beta =1, a = 0.5, b = 0.9, d = 0.3$. (**g**) One equilibrium point for $l= .1, \beta =5, a = 10.5, b = 5.2, d = 0.3$. (**h**) For $l= 0.2; \beta =10.7; a = 7.5; b = 4.1; d = 0.7$, there exists no interior equilibrium point.

**Figure 2:**
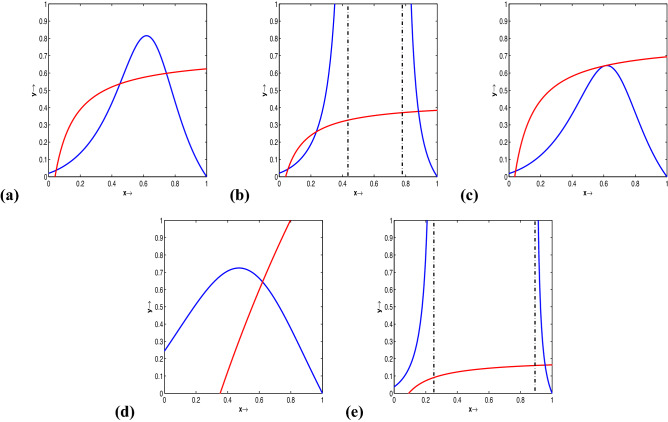
(Colour online) Weak Allee effect: prey-nullclines are identified by solid blue curves and, accordingly, predator-nullclines are recognized by solid red curves. (**a**) For $l= -0.2, \beta =10.7, a = 10.9, b = 3.2, d = 0.3$, both the nullclines cross thrice, suggesting that there are three interior equilibrium points. (**b**) Three interior equilibrium points exist for $l= -0.2, \beta =10.7, a = 10.9, b = 5.2, d = 0.3$. (**c**) For $l= -0.2, \beta =11.494054912906, a = 10.9, b = 3.2, d = 0.3$, the two of the equilibria collide and consequently, there exist two interior equilibrium points. (**d**) One equilibrium exists for $l= -0.2, \beta =1, a = 0.5, b = 0.9, d = 0.3$. (**e**) One equilibrium point exists for values $l= -0.2; \beta =6.4, a = 10.5, b = 5.2, d = 0.3$.

The system (10) has the following equilibria:

(i) $E_0\equiv (0,0)$, the trivial equilibrium,
(ii) $E_1\equiv (1,0)$, the first axial equilibrium,
(iii) $E_2\equiv (l,0)$, the second axial equilibrium. Existence and the multiplicity of positive equilibria depends upon the shapes and positions of the nontrivial nulclines and the parametric restrictions. If $E_{3}\equiv (x^*, y^*)$ is positive equilibrium hence $y^*=\frac{\beta x^*-(1+ax^*)d}{bd(1+ax^*)}$, with $\beta > ad$, where $x^*$ is a positive root of the biquadratic equation
  11 $$\begin{aligned} Q(x) \equiv k_4x^4+k_3x^3+k_2x^2+k_1x+k_0=0, \end{aligned}$$
  and the coefficients are $k_4=ab>0$, $k_3=b\{1-a(1+l)\}$, $k_2=-b(1+l)+lab$, $k_1=lb+\beta -ad$, $k_0=-d<0$.

Examples of mutual positions of the prey-nullcline and predator-nullcline are presented in Fig. 1 (for Strong Allee effect) and Fig. 2 (for Weak Allee effect). The utilized values used in Figs. 1 and 2 are provided in the corresponding figure captions.

### Stability in absence of diffusion

In this section, we investigate the local behavior of the equilibria next to the existence of Hopf bifurcation in the case of the non spacial model.

At $E_0 (0,0)$ The Jacobian matrix has eigenvalues strictly negative (which are $-l$ and $-d$), thus $E_0$ is stable in nature.

At $E_1 (1,0)$ the Jacobian matrix has eigenvalues $l-1$ and $\frac{\beta }{1+a}-d$, thus $E_1$ is stable node if $\beta < d(1+a)$ otherwise it is a saddle point.

At $E_2 (l,0)$ the Jacobian matrix has eigenvalues $-l(1-l)$ and $\frac{\beta l}{1+al}-d$, thus $E_2$ is stable node if $\beta l< d(1+al)$ otherwise it is a saddle point^47^.

Now we calculate the Jacobian matrix $J_i^*$ of (8) at $E_i^*$ we obtain

12 $$\begin{aligned} J_i^* = \left[ \begin{array}{cc} x_i(1+l-2x_i)-\frac{\beta ax_i y_i}{(1+ax_i)^2(1+by_i)} &{}-\frac{\beta x_i}{(1+ax_i)(1+by_i)^2}\\ \frac{\beta y_i}{(1+ax_i)^2(1+by_i)}&{} -\frac{\beta bx_iy_i}{(1+ax_i)(1+by_i)^2} \end{array}\right] , \quad i=1,2,3. \end{aligned}$$

With the help of Routh–Hurwitz stability criterion $E_{i}^{*}$ will be locally stable if tr$J_{i}^{*}<0$ and det$J_{i}^{*}>0$. Mathematically, one may conclude that the equilibrium point $E_{i}^{*}$ of the proposed system will be locally stable if the following conditions hold:

(i) $$x_i(1+l-2x_i)-\frac{\beta x_i y_i}{(1+ax_i)(1+by_i)} \bigg (\frac{a}{1+ax_i}+\frac{b}{1+by_i}\bigg ) < 0$$,
(ii) $$\frac{(1+abx_{i} y_{i})\beta ^{2}x_{i} y_{i}}{(1+ax_i)^{3}(1+by_i)^{3}}-\frac{\beta bx_{i}^{2}(1+l-2x_i)y_{i}}{(1+ax_i)(1+by_i)^2} > 0$$.

For a certain parameter value, the qualitative change of dynamics occurs in the system and that critical parameter value is called bifurcation point. Thus, in order to identify the possible qualitatively different dynamical behaviour, we now investigate the possibility for Hopf bifurcation at the interior as well as co-existence equilibrium point $E_{3}$ by taking $\beta$ as bifurcating parameter and keeping other parameters fixed.

In general, Hopf bifurcation takes place in a two dimensional (2D) model system as a spiral point exchanges from unstable to stable or, vice-versa and a periodic solution disappears or, emerges. It is interesting to note that we have paid our attention to the system Hopf bifurcation around $E_{3}$ only as $E_{3}$ is an interior as well as stable co-existence equilibrium point for a specific set parameters, but other equilibria are either axial or unstable for the said definite numerical simulation. The next theorem investigates the existence of Hopf bifurcation for the system (8).

#### Theorem 3.1

*The system* (8) *undergo Hopf bifurcation at* $\beta =\beta _{c}$ *around* $E_{3}$ *if*

(i) $$\left[ tr(J_{E_{3}})\right] _{\beta =\beta _{c}}=0$$,
(ii) $$\left[ det(J_{E_{3}})\right] _{\beta =\beta _{c}}>0$$ *and*
(iii) $$\left[ \frac{d (\textrm{tr}(J_{E_{3}})}{d \beta }\right] _{\beta =\beta _{c}} \ne 0$$.

#### Proof

The characteristic equation of $J_{3}$ at $E_{3}$ is

$$\begin{aligned} \lambda ^2-{\mathrm{{tr}}}(J_{E_{3}})\lambda + {\mathrm{{det}}}(J_{E_{3}})=0, \end{aligned}$$

For the Hopf bifurcation at interior equilibrium $E_{3}$, the quadratic equation must have a pair of purely imaginary roots. we consider a crucial value of $\beta$, say $\beta _{c}$, $\left[ tr(J_{E_{3}})\right] =0$ and in this case, the quadratic equation becomes

$$\begin{aligned} \lambda ^{2}+ \left[ {\textrm{det}}(J_{E_{3}})\right] _{\beta =\beta _{c}}=0, \end{aligned}$$

has a roots $\lambda _{1,2}=\pm i\omega _0$, where $\omega _0=\sqrt{\left[ \textrm{det}(J_{E_{3}})\right] _{\beta =\beta _{c}}}$. To check the transversality condition, we consider $\beta$ in the neighborhood of $\beta _c$, $\lambda _{1,2}=\mu (\beta )\pm i\omega (\beta )$, then $\mu (\beta )=\frac{1}{2}\textrm{tr}(J_{E_{3}})$ and $\omega (\beta )=\sqrt{\left[ \textrm{det}(J_{E_{3}})-\frac{1}{4}(\textrm{tr}(J_{E_{3}})^2\right] }$. Now,

$$\begin{aligned} \left[ \frac{d \mu (\beta )}{d \beta }\right] _{\beta =\beta _{c}}= & {} \frac{1}{2} \left[ \frac{d (\textrm{tr}(J_{E_{3}})}{d \beta }\right] _{\beta =\beta _{c}}\\= & {} \frac{1}{2} \left[ -\frac{ax_2y_2}{(1+ax_2)^2(1+by_2)}-\frac{bx_2y_2}{(1+ax_2)(1+by_2)^2}\right] _{\beta =\beta _{c}}\\\ne & {} 0. \end{aligned}$$

Thus, the system undergoes Hopf bifurcation at $\beta =\beta _{c}$. $\square$

**Figure 3:**
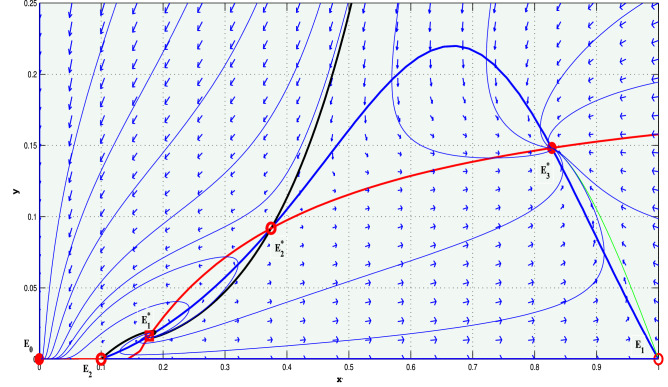
(Strong Allee effect): this figure represents phase portraits of (8) with three positive equilibria. The deep blue curve is the prey-nullcline and the curve presented in deep red is the predator-nullcline. The attractor equilibria are $E_0$ and $E_{3}$, saddle equilibria are $E_1$, $E_2$ and $E_2^*$, unstable focus $E_1^*$ are highlighted with filled circles, open circles and square open box, respectively. The stable manifolds of the saddle equilibria ($E_2$ and $E_2^*$), called the separatrix curves, are highlighted by solid black curves. $E_0$ and $E_{3}$ are separated by those two stable manifolds of saddle interior equilibrium point $E_2$ and $E_{2}^{*}$, both of which are drawn with black curves. The parameter values used are given by $l = 0.1, a = 7.8, b = 4.1, d= 0.7, \beta = 9.8$.

**Figure 4:**
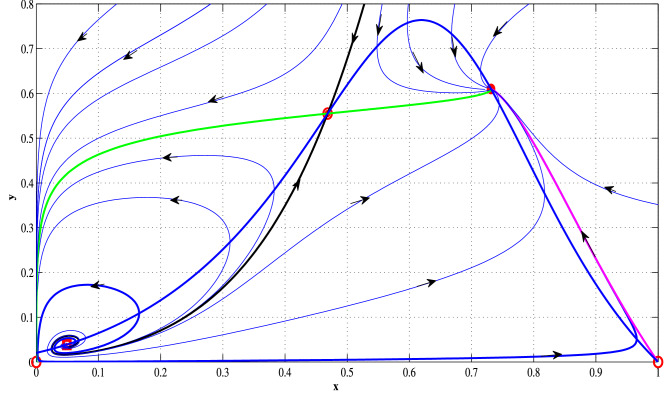
(Weak Allee effect): this figure represents phase portraits of (8) with three positive equilibria.The attractor equilibria are $E_0$ and $E_{3}$, three saddle ($E_1$, $E_2$ and $E_{2}^{*}$) and one unstable focus $E_1^*$ are shown with filled circles, open circles and square open box, respectively. The stable manifolds of the saddle equilibria ($E_{2}$ and $E_{2}^{*}$), called the separatrix curves, are presented by solid black curves. $E_0$ and $E_{3}$ are separated by those two stable manifolds of saddle interior equilibrium point $E_2$ and $E_{2}^{*}$, both of which are drawn with black curves. The parameter values used are given by $l = -2, a = 11, b = 3, d= 0.3, \beta = 10.5$.

**Figure 5:**
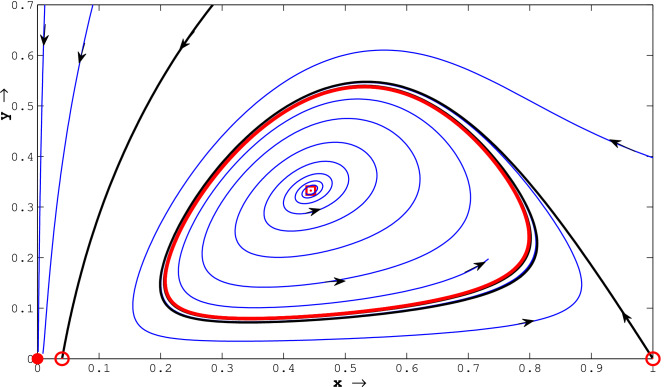
(Strong Allee effect:) the fixed point(0, 0) is stable (shown by filled circle) whereas (0.04, 0) and (1, 0) are saddle points (shown by open circles). The single interior fixed point is unstable and shown with a square open box. This unstable fixed point is surrounded by a stable limit cycle which is shown as a thick red curve. The limit cycle attracts the neighboring trajectories. The stable manifold of (0.04, 0), shown by black curve passing through (0.04, 0), acts as a separatrix curve that splits the dynamics of trajectories of the model into two regions in the phase plane; the trajectories initiated from the region left to this black curves enter (0, 0) and the basins of attraction of the stable limit cycle is the domain right to it. The other black curve through (1, 0) is unstable manifold of the fixed point(1, 0). The parameter values used for this figure are $l=0.04; \beta =1.1;a=0.5;b=1.0; d=0.3$.

**Figure 6:**
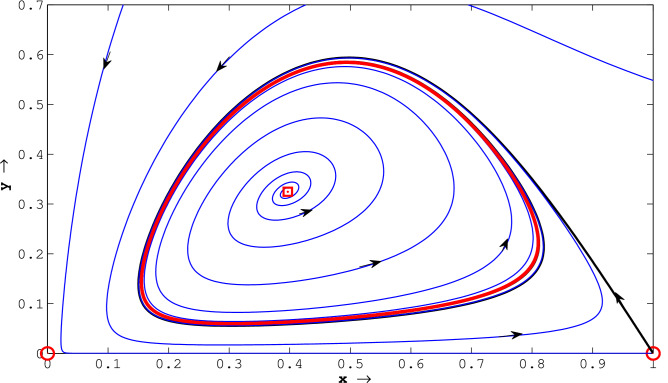
(Weak Allee effect:) the fixed points (0, 0) and (1, 0) are saddle points. These are shown with open circles. The single interior fixed point is locally unstable and shown with a square open box. This unstable equilibrium is surrounded by a stable limit cycle which is shown as a thick red curve. The limit cycle attracts the neighboring trajectories. The stable and unstable manifolds of (0, 0) are *y* and *x* axis, respectively. The stable manifold of (1, 0) is the *x* axis and its unstable manifold is shown by black curve. The considered parameters are $l=-0.01; \beta =1.2;a=0.5;b=1.0; d=0.3$.

### Spatiotemporal patterns generated by the presence of spatial diffusion

In the following discussions, we will continue to limit ourselves to the case that the non-spatial system of (9) has unique, coexistence steady state $E_{3} (x^*, y^*)$. The unique positive equilibrium $E_{3}$ for the diffusive model of (9) is a homogeneous steady state for the diffusive system (9). Under certain parametric restrictions, the diffusion can generate instability of this equilibrium, which means that this equilibrium can stable in the absence of diffusion, and in the presence of this last it become unstable, this is the familiar observable fact of Turing instability^34^.

Diffusion-driven instability is found in interacting species system which can present a rich dynamics for ecosystems. local stability analysis at $E_{3}$ for the diffusive system (9) and the required necessary and sufficient conditions for having diffusion-driven instability are well-known. To perform a linear stability analysis around the co-existence equilibrium point $E_{3}$, one must linearized the diffusive system (9) about the spatially homogeneous steady state $E_{3}(x^{*}, y^{*})$, we assume that the solution are expressed in the following structure:

13 $$\begin{aligned} \left[ \begin{array}{cc} x\\ y\end{array} \right]= & {} \left[ \begin{array}{cc} x^{*}\\ y^{*}\end{array} \right] + \epsilon \left[ \begin{array}{cc} x_{k}\\ y_{k}\end{array} \right] e^{\mu t+i (k_{\xi }\xi +k_{\eta }\eta )}+c.c.+{\mathscr {O}}(\epsilon ^{2}), \end{aligned}$$

where $\mu$ is the growth rate of perturbation in time *t*; $k\;(=\sqrt{k_{\xi }^{2}+k_{\eta }^{2}})$ is the wave number and c.c. stands for complex conjugate. Substituting (13) into (9) and neglecting all non-linear terms in *x* and *y*, we obtain

14 $$\begin{aligned} (J_{11}-k^{2}d_{1}-\mu )(J_{22}-k^{2}d_{2}-\mu )-J_{12} J_{21}=0, \end{aligned}$$

Consequently, the solution of (14) is

$$\begin{aligned} \mu _{k}= & {} \frac{-\alpha _{k} \pm \sqrt{\alpha _{k}^{2}-4\beta _{k}}}{2}, \\ \alpha _{k}= & {} k^{2}(d_{1}+d_{2})-(J_{11}+J_{22}), \\ \beta _{k}= & {} d_{1}d_{2} k^{4}- (d_{1}J_{22}+d_{2}J_{11})k^{2} + (J_{11}J_{22}-J_{12}J_{21}). \end{aligned}$$

One can easily observe that if one of the roots ($\mu _{k}$) of (14) is positive then the reaction–diffusion system (9) will be unstable.

On a square 2*D* domain with Neumann boundary condition, Turing instability requires the following conditions, see^35,48–52^ for details:

$$\begin{aligned}{} & {} (i)\;J_{11}+J_{22} < 0,\\{} & {} (ii)\;J_{11} J_{22}-J_{12} J_{21}> 0,\\{} & {} (iii)\;d_{1}J_{22}+d_{2}J_{11}> 0,\\{} & {} (iv)\;\frac{(d_{1}J_{22}+d_{2}J_{11})^{2}}{4d_{1}d_{2}}> (J_{11} J_{22}-J_{12} J_{21}),\\{} & {} \quad \Rightarrow d_{1}J_{22}+d_{2}J_{11} > 2 \sqrt{d_{1}d_{2}(J_{11} J_{22}-J_{12} J_{21})}, \end{aligned}$$

where the Jacobian matrix *J* of (9) is given by

$$\begin{aligned} J = \left[ \begin{array}{cc} x^*(1+l-2x^*)-\frac{\beta ax^*y^*}{(1+ax^*)^2(1+by^*)} &{}-\frac{\beta x^*}{(1+ax^*)(1+by^*)^2}\\ \frac{\beta y^*}{(1+ax^*)^2(1+by^*)}&{} -\frac{\beta bx^*y^*}{(1+ax^*)(1+by^*)^2} \end{array}\right] =\left[ \begin{array}{cc} J_{11}&{} J_{12}\\ J_{21} &{} J_{22}\end{array} \right] . \end{aligned}$$

(i) and (ii) are the Routh–Hurwitz criterion for which $E_{3}$ corresponding to the non-spatial model of (9) is linearly stable. It is interesting to note that the last condition (*iv*) encapsulates condition (*iii*) as well. Also, one can observe that the three constraints (*i*), (*ii*), (*iv*) are required conditions for Turing pattern formation given the proper perturbation and system length scale^53,54^. The parametric space which satisfies (*i*)–(*iv*) is known as as ‘Turing space’.

## Numerical simulations

Plotting non-trivial nullclines with various parameter sets, we have seen that the system has three, two, one or no positive equilibriums in the first quadrant for both strong and weak Allee effect cases. The associated parameter sets used for the simulation are shown in the captions of Figs. 1 and 2. The phase portraits of (8) with three positive equilibrium points are presented with parameter values used are given by $l = 0.1, a = 7.8, b = 4.1, d = 0.7, \beta = 9.8$ (for strong Allee effect) and $l = -2, a = 11, b = 3, d = 0.3, \beta = 10.5$ (for weak Allee effect). The stable manifolds of the saddle equilibria, called the separatrix curves, are shown by solid black curves in Figs. 3 and 4. The attractive basins of trivial equilibrium points (0, 0) and one attractor positive equilibrium are separated by stable manifolds of saddle positive equilibrium points, drawn with black curves are illustrated therein. For the parameter sets $l=0.04; \beta =1.1;a=0.5;b=1.0; d=0.3$, (for strong Allee effect: cf. Fig. 5) and $l=-0.01; \beta =1.2; a=0.5; b=1.0; d=0.3$, (for weak Allee effect: cf. Fig. 6), one can observe that there exist unique interior fixed points being locally unstable, and the said fixed points are surrounded by unique stable limit cycles. For details, see the captions of the figures mentioned herein.

### Turing pattern formation with Allee effects

This subsection investigates with the extensive numerical illustrations of (9) with the inclusion of Allee effect and explain qualitative results in 2*D* space. The numerical integration of The model system (9) is numerically solved by means of an explicit Euler method for the time with the step 0.01; size of step space is 1.0; measure of domain is $200 \times 200$ and the classical approximation of boundary condition. The initial data are a small random perturbation about the co-existence equilibrium point $E_{3}(x^{*}, y^{*})$.

**Figure 7:**
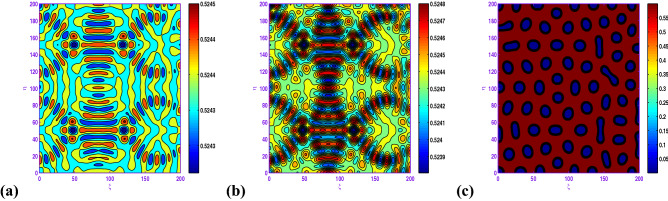
Transition of spatial dynamics of the prey population (*u*) in the model (9) , for the set $a=0.5$, $b=0.9$, $\beta =1.0$, $d=0.3$, $l=0.01$, $d_{1}=0.1$, $d_{2}=20.0$ at (**a**) $t=1000$; (**b**) $t=2000$; (**c**) $t=5000$.

Figure 7 represents the process of spatial pattern for (9) with $a=0.5$, $b=0.9$, $c=1.0$, $d=0.3$, $l=0.01$, $d_{1}=0.1$ and $d_{2}=20.0$. In this case, the pattern takes a long time stabilize at a formation of holes and short stripes (cf. Fig. 7c) at stationary level.

**Figure 8:**
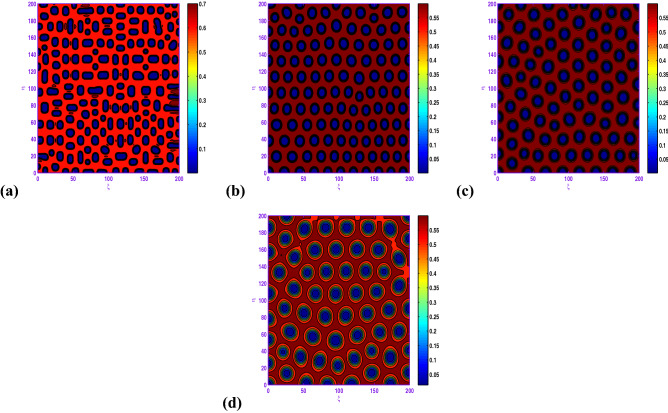
Transition of spatial dynamics of the prey population (*u*) in the model (9) at $t=5000$, for the set: $a=0.5$, $b=0.9$, $\beta =1.05$, $d=0.3$, $l=0.01$, $d_{2}=20.0$ and (**a**) $d_{1}=0.05$; (**b**) $d_{1}=0.1$; (**c**) $d_{1}=0.2$; (**d**) $d_{1}=0.5$.

Clearly, the results obtained by Fig. 8 that when $d_{1}$ is increased from 0.05 to 0.5, there exhibits a transition from almost holes pattern (cf. Fig. 8a) to the holes pattern with larger radius (cf. Fig. 8d). Also, there is an important observation that when *c* is changed from 1.0 to 1.05, there exhibits a transition from hole and short-stripe mixtures (cf. Fig. 7c) to the holes (cf. Fig. 8b) corresponding to $a=0.5$, $b=0.9$, $d=0.3$, $l=0.01$, $d_{1}=0.1$ and $d_{2}=20.0$.

**Figure 9:**
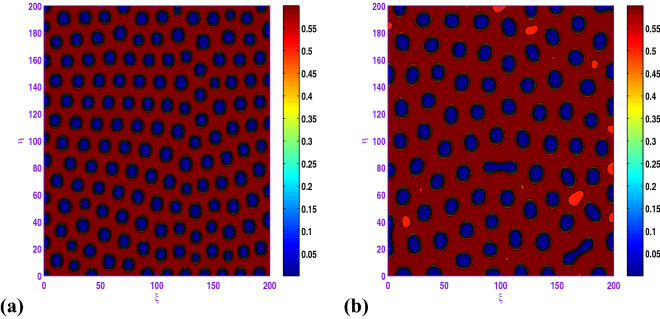
Transition of spatial dynamics of the prey population (9) at $t=5000$, for the set $a=0.5$, $b=0.9$, $\beta =1.05$, $d=0.3$, $d_{1}=0.1$, $d_{2}=20.0$ and (**a**) $l=-0.01$; (**b**) $l=-0.04$.

When $l=-0.01$, we show the holes pattern of the prey population at $t=5000$ in Fig. 9a corresponding to $a=0.5$, $b=0.9$, $c=1.05$, $d=0.3$, $d_{1}=0.1$, $d_{2}=20.0$. From the obtained illustrations in Figs. 8b and 9a are similar, there is an radical change for the spatial modes. Finally, we uncover that the hole patterns coexist with the short-stripe patterns for $l=-0.04$ in the spatial model, and the system behavior of the reaction–diffusion model does not experience any more modifications.

**Figure 10:**
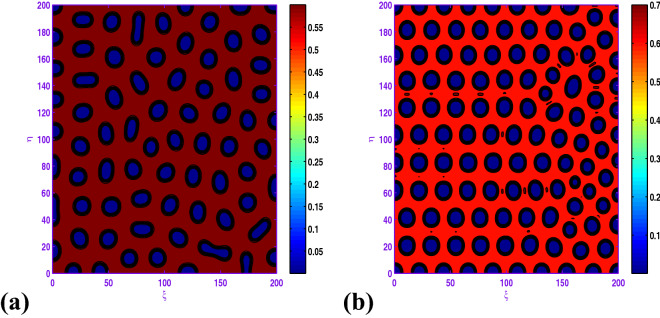
Transition of spatial dynamics of the prey population (*u*) in the model (9) at $t=5000$, for the set: $a=0.5$, $b=1.0$, $\beta =1.05$, $d=0.3$, $d_{1}=0.1$, $d_{2}=20.0$ and (**a**) $l=-0.01$; (**b**) $l=0.04$.

In Fig. 10, with $l=-0.01$ or $l=0.04$, the system dynamics undergos a transition from hole and short-stripe mixtures growth to hole replication; that is, short-stripes decay and the holes pattern emerges. Moreover, from these two panels of Fig. 10, one can observe that as *l* increases from $l=-0.01$ to $l=0.04$, the prey concentration changes to high for the set $a=0.5$, $b=1.0$, $c=1.05$, $d=0.3$, $d_{1}=0.1$, $d_{2}=20.0$.

**Figure 11:**
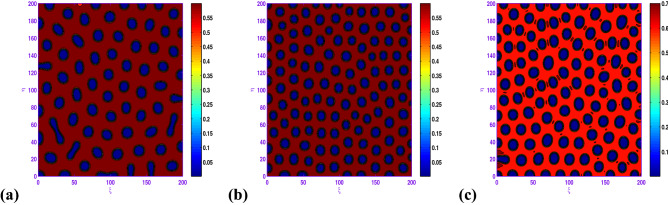
Transition of spatial dynamics of the prey population (*u*) in the model (9) at $t=5000$, for the set: $a=0.6$, $b=0.98$, $\beta =1.1$, $d=0.3$, $d_{1}=0.1$, $d_{2}=20.0$ and (**a**) $l=-0.03$; (**b**) $l=0.0$; (**c**) $l=0.025$.

If we consider $a=0.6$, $b=0.98$, $c=1.1$, $d=0.3$, $d_{1}=0.1$, $d_{2}=20.0$ with some values of *l*, one can observe from Fig. 11 that the hole and short-stripe patterns coexist for $l=-0.03$ and as the value of *l* increases ($l=0.0, 0.025$) regular hole is observed. It is curiously to remark from the results about of this figure that both bounds of the prey population over 2*D* domain are changing in terms of *l* (cf. Fig. 11b,c).

### Turing pattern formation without Allee effects

Here, we explore the impact of disperse on the spatiotemporal patterns without Allee effects of the following reaction–diffusion predator–prey model:

15a $$\begin{aligned} \frac{\partial x}{\partial t}= & {} x\left( 1-x\right) -\frac{\beta xy}{1+ax+by+abxy} + d_{1}\nabla ^{2}x, \end{aligned}$$

15b $$\begin{aligned} \frac{\partial y}{\partial t}= & {} \frac{\beta xy}{1+ax+by+abxy}-dy + d_{2}\nabla ^{2}y, \end{aligned}$$

15c $$\begin{aligned} x(0, \xi , \eta )\ge & {} 0, \; \; y(0, \xi , \eta ) \ge 0. \end{aligned}$$

**Figure 12:**
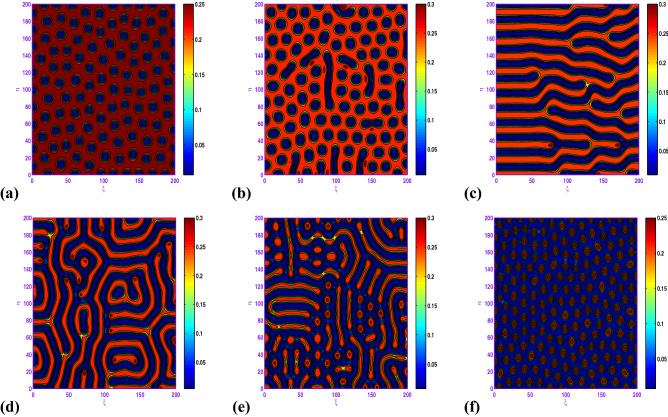
Transition of spatial dynamics of the prey population (*u*) in the model (15) at $t=5000$, for the set: $a=2.1$, $b=3.01$, $d=0.2$, $d_{1}=0.1$, $d_{2}=20.0$ and (**a**) $\beta =4.8$; (**b**) $\beta =4.9$; (**c**) $\beta =5.1$; (**d**) $\beta =5.2$; (**e**) $\beta =5.4$; (**f**) $\beta =6.0$.

**Figure 13:**
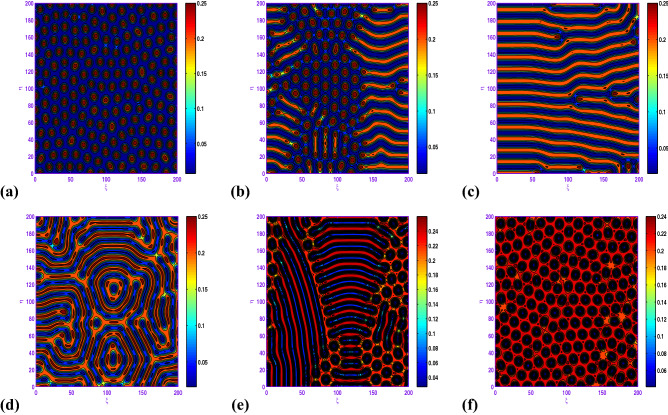
Transition of spatial dynamics of the prey population (*u*) in the model (15) at $t=5000$, for the set: $a=2.1$, $b=3.01$, $\beta =6.0$, $d_{1}=0.1$, $d_{2}=20.0$ and (**a**) $d=0.2$; (**b**) $d=0.24$; (**c**) $d=0.261$; (**d**) $d=0.3$; (**e**) $d=0.32$; (**f**) $d=0.34$.

On increasing the numerical value of control parameters $\beta$ and *d*, one may observe from Figs. 12 to 13 that the pattern sequence “holes (cf. Fig. 12a) $\rightarrow$ hole-stripe mixtures (cf. Fig. 12b) $\rightarrow$ stripes (cf. Fig. 12c) $\rightarrow$ labyrinthine (cf. Fig. 12d) $\rightarrow$ spot-stripe mixtures (cf. Fig. 12e) $\rightarrow$ spots (cf. Fig. 12f)“ and “spots (cf. Fig. 13a) $\rightarrow$ spot-stripe mixtures (cf. Fig. 13b) $\rightarrow$ stripes (cf. Fig. 13c) $\rightarrow$ labyrinthine (cf. Fig. 13d) $\rightarrow$ hole-stripe mixtures (cf. Fig. 13e) $\rightarrow$ holes (cf. Fig. 13f)” are observed respectively. From ecological standpoint one may notice that the cold spots pattern confirms that the prey species are motivated by predators to a very low level in those reaction–diffusion domains (i.e. predator is pre-dominant in the domain), whereas the hot spots pattern confirms that the prey are motivated by predators to a high level in those reaction–diffusion domains (that is prey is pre-dominant in the domain).

## Discussion and conclusion

It is a well-documented fact that the Allee effects are widely recognized as one of the most influential decisive factors in population dynamics. The present study is based on a predator–prey interaction with a single Allee effect in the growth of the prey population. The generic Crowley–Martin type response function for the predator population is taken into consideration in the investigated system. Despite exploring the dynamics of the interacting species influenced by the Allee effect on prey, considerable effort is made to uncover how to reduce the adverse effects by altering other system parameters. The dynamical system of concern reveals its complexity in terms of bi-stability and oscillatory phenomena. The bi-stability records that a slight change of initial conditions may lead to significant consequences even to the extent of extinction of both species and coexistence, while oscillations bespeak that small modifications of parameters may result in enormous effects on the asymptotic behavior of the model. The proposed model’s stability and Hopf bifurcation conditions have been derived analytically. From the ecological interest, the existence of a unique limit cycle eventuating from Hopf bifurcation confirms both species’ oscillatory features. All the results derived analytically are validated through graphical representations of the present model under contemplation.

In the realm of the ecological system, the disposition of stationary spatial patterns generated by Turing instability has been the subject of present-day research. The present article on the spatiotemporal dynamics with insertion of Allee effect on prey species together with the use of Crowley–Martin response function is studied quantitatively. Following numerical simulations based on mathematical analysis, various spatial patterns appear to emerge depending on the range of model parameters in the Turing space. One may record the interesting observation that the spatiotemporal model dynamics expose diffusion-controlled growth formation not only to holes, stripes, and spots alone but also to labyrinthine replication. Figures 7, 8, 9, 10 and 11 *exhibit several panels of spatial patterns that emerged as short-type or hole or coexistence of both in particular. Moreover, the numerical simulations in the absence of Allee effect reveal the sequence of spatial patterns “holes $\rightarrow$ hole-stripe mixtures $\rightarrow$ stripes $\rightarrow$ labyrinthine $\rightarrow$ spot-stripe mixtures $\rightarrow$ spots ” or “spots $\rightarrow$ spot-stripe mixtures $\rightarrow$ stripes $\rightarrow$ labyrinthine $\rightarrow$ hole-stripe mixtures $\rightarrow$ holes ” as evident from Figs. 12 to 13. The results displayed in a nutshell clearly highlighted the crucial role played by the Allee effect together with diffusion in pattern formation of the proposed Crowley–Martin predator–prey model in order to establish their own importance in the realm of an ecological system.

The eminence of the present investigation lays out a step ahead towards providing the complex dynamics of a rational predator–prey interaction to the community of theoretical ecology at large. Any befitting future development relating to the domain of concerned research can be established successfully by resorting to the actual findings so that the updated system continues to find outcomes closer to the real situation. Future advancement towards forming more complex spatio-temporal models with the inclusion of the Allee effect with time-delay, noise reflection, travelling wave propagation, food chain models, etc., should certainly be some uncovered areas of the essence. It would undoubtedly be more challenging to explore the non-local and co-dimension-two bifurcation phenomena; hence, further discussion is withdrawn in advance.

It is really challenging to fit the actual data into mathematical systems of interacting species with the Allee effect that provides the best assessment for population conservation. Conversely, it needs precise data on population in space and time so as to validate the model’s predictions prepared by ecologists. Alternatively, one needs to develop more sophisticated spatial models and modern mathematical methods to quantify observed spatial patterns comprehensively. In summary, collaborative attempts from ecologists, mathematicians, and statisticians to carry out an in-depth study on spatiotemporal dynamics would significantly contribute to the domain under consideration. It would always be better to comply with mathematical modelling in ecology called for biodiversity, conservation, and management procedures through successful collaborations in the days to come^55^.

## Data Availability

The datasets used during the current study are available from the corresponding author upon reasonable request.
